# Deep Sequencing of the *Scutellaria baicalensis* Georgi Transcriptome Reveals Flavonoid Biosynthetic Profiling and Organ-Specific Gene Expression

**DOI:** 10.1371/journal.pone.0136397

**Published:** 2015-08-28

**Authors:** Jinxin Liu, Jingyi Hou, Chao Jiang, Geng Li, Heng Lu, Fanyun Meng, Linchun Shi

**Affiliations:** 1 Key Laboratory of Traditional Chinese Medicine Research and Development of Hebei Province, Institute of Traditional Chinese Medicine, Chengde Medical University, Chengde, 067000, China; 2 Beijing Area Major Laboratory of Protection and Utilization of Traditional Chinese Medicine, Beijing Normal University, Beijing, 100875, China; 3 State Key Laboratory Breeding Base of Dao-di Herbs, China Academy of Chinese Medical Sciences, Beijng, 100700, China; 4 Institute of Medicinal Plant Development, Chinese Academy of Medical Sciences, Peking Union Medical College, Beijing, 100193, China; National Institute of Plant Genome Research, INDIA

## Abstract

*Scutellaria baicalensis* Georgi has long been used in traditional medicine to treat various such widely varying diseases and has been listed in the Chinese Pharmacopeia, the Japanese Pharmacopeia, the Korean Pharmacopoeia and the European Pharmacopoeia. Flavonoids, especially wogonin, wogonoside, baicalin, and baicalein, are its main functional ingredients with various pharmacological activities. Although pharmaological studies for these flavonoid components have been well conducted, the molecular mechanism of their biosynthesis remains unclear in *S*. *baicalensis*. In this study, Illumina/Solexa deep sequencing generated more than 91 million paired-end reads and 49,507 unigenes from *S*. *baicalensis* roots, stems, leaves and flowers. More than 70% unigenes were annotated in at least one of the five public databases and 13,627 unigenes were assigned to 3,810 KEGG genes involved in 579 different pathways. 54 unigenes that encode 12 key enzymes involved in the pathway of flavonoid biosynthesis were discovered. One baicalinase and three baicalein 7-O-glucuronosyltransferases genes potentially involved in the transformation between baicalin/wogonoside and baicalein/wogonin were identified. Four candidate 6-hydroxylase genes for the formation of baicalin/baicalein and one candidate 8-O-methyltransferase gene for the biosynthesis of wogonoside/wogonin were also recognized. Our results further support the conclusion that, in *S*. *baicalensis*, 3,5,7-trihydroxyflavone was the precursor of the four above compounds. Then, the differential expression models and simple sequence repeats associated with these genes were carefully analyzed. All of these results not only enrich the gene resource but also benefit research into the molecular genetics and functional genomics in *S*. *baicalensis*.

## Introduction

*Scutellaria baicalensis* Georgi (Baikal skullcap or Huang-Qin in Chinese), belonging to the Lamiaceae family, is widely used in traditional medicine and has been listed in the Chinese Pharmacopeia [1], the Japanese Pharmacopeia [2], the Korean Pharmacopoeia [3] and the European Pharmacopoeia [4]. Its dry root that contained multiple flavone derivatives has been widely used to treat a variety of diseases like cancer, hepatitis, allergies, inflammation, skin conditions, and epilepsy [5]. Baicalin, baicalein, wogonoside and wogonin are the main flavonoid components in *S*. *baicalensis*, and have various pharmacological activities, such as antitumor effects, antioxidative action, anti-inflammatory, antibacterial and antiviral activities [6–8]. Sho-Saiko-To, a Japanese herbal supplement believed to enhance liver health, includes baicalin, baicalein, and wogonin as the major active ingredients [9], Baicalin is the glucuronide of baicalein, whereas wogonoside is the glucuronide of wogonin. Baicalin and wogonoside can be hydrolyzed into baicalein and wogonin directly [10]. The amount of total baicalein component in roots of *S*. *baicalensis* is far higher than that in the aboveground parts including stems, leaves and flowers. With the dramatically increasing utilization of *S*. *baicalensis* for medicine in recent years, the wild resource of the plant is too limited to satisfy demand.

Flavonoids comprise a large group of secondary metabolities widely distributed in the plant kingdom that share the same 15-carbon basic skeleton (C6-C3-C6), consisting of two phenyl rings (A and B) and heterocyclic ring (C). Flavonoid synthesis starts with the condensation of three molecules malonyl-CoA with one molecule *p*-coumaroyl-CoA to a chalcone intermediate, which carrying out by the enzyme chalcone synthase (K00660, CHS)[11]. The two immediate precursors 4-coumaroyl-CoA and malonyl-CoA originate from two primary metabolism pathways known as the general phenylpropanoid pathway and the Krebs tricarboxylic acid cycle [12]. The chalcone is subsequently conjugated ring-closure to form the three-ringed structure of a flavanone by the enzyme chalcone flavanone isomerase (K01859, CHI)[13]. From these central intermediates the metabolic pathway continues through several side branches to transform various flavonoids, including the flavonols, anthocyanidins, flavan-3-ols, isoflavones, et al. [14]. The known enzymes for these transformation are: naringenin 3-dioxygenase (K00475, F3H), flavonol synthase (K05278, FLS)[15], trans-cinnamate 4-monooxygenase (K00487, CYP73A)[16], Polyketide reductase (K08243, PKR)[17], flavonoid 3'-monooxygenase (K05280, E1.14.13.21)[18], leucoanthocyanidin dioxygenase (K05277, E1.14.11.19), anthocyanidin reductase (K08695, ANR)[19], leucoanthocyanidin reductase (K13081, LAR)[20], shikimate O-hydroxycinnamoyltransferase (K13065, HCT)[21], coumaroylquinate (coumaroylshikimate) 3'-monooxygenase (K09754, C3'H)[22], flavanone 4-reductase (K13082, DFR)[23], caffeoyl-CoA O-methyltransferase (K00588, E2.1.1.104), flavonoid 3',5'-hydroxylase (K13083, CYP75A)[24], et al. The chalcone synthase (CHS) has been isolated from hairy root cultures of *S*. *viscidula* by rapid amplification of cDNA ends (RACE)[25]. And, the levels of flavone can be enhanced through overexpression of chalcone isomerase in hairy root cultures of *S*. *baicalensis* [26]. The cDNA sequences of Phenylalanine ammonia-lyase (PAL), naringenin 3-dioxygenase (F3H), flavanone 4-reductase (DFR) has also been determined in *S*. *viscidula*[27].

Baicalin, baicalein, wogonoside and wogonin are type of flavones. Flavones are synthesized at a branch point of the anthocyanidin/proanthocyanidin pathway and flavanones are known as its direct precursor. Excepted in the Apiaceae family, flavone formation in various tissues of a wide range of higher and lower plant species is catalyzed by the FNS II[28]. The 6-OH is the most important characteristic of baicalin and baicalein. The 6-C hydroxylation is catalyzed by the flavonoid 6-hydroxylase (F6H) in *Glycine max*. Flavonoid 6-hydroxylas catalyzed the conversion of flavanones more efficiently than flavones, and hardly for isoflavones hydroxylated [29]. Wogonoside and wogonin have the additional 8-methoxy and absent of 6-hydroxy substitution. 0-Methylation of flavonoid compounds has been shown to be catalyzed by position-specific 0-methyltransferases[30]. Flavonol 8-O-methyltransferase catalyzing the transfer of the methyl group of S-adenosyl-L-methionine to the 8-hydroxyl group of flavonols was purified from *Lotus corniculatus* [31]. Baicalin and wogonoside are the glucosylation of baicalein and wogonin in the 7-O-position. The enzyme catalyzes the transfer of the glucosyl moiety from UDP-sugar to the 7-O-position of flavonoids by favonoid 7-O-glucosyltransferase[32], which has been cloned from hairy root cultures of *S*. *baicalensis*[33]. In addition, a flavonoid glycosyltransferase (SbUGT) from *S*. *barbata* is identified as another efficient flavonoid glucosyltransferase using various flavonoids as substrates[34].

Transcriptome profiling is an important tool for understanding active component biosynthesis at the transcriptional level [35]. At present, microarray and RNA sequencing (RNA-seq) constitute the two most popular methods that are employed for genome-wide transcriptome profiling. The application of microarray for gene expression analysis was limited by background hybridization, known sequencing information and comparability [36]. Next-generation sequencing technologies constitute a recently developed, sequence-based method that has revolutionized traditional sequencing and has been regarded as a new platform to replace microarray [37]. RNA-seq strategy was developed to monitor transcriptomic dynamics using next-generation, deep-sequencing technologies [38]. In brief, messenger RNA is converted to cDNA fragments with adaptors that are attached to one or both ends. After PCR amplification, the library is sequenced to obtain short reads that are either aligned to a reference genome or transcripts, or assembled *de novo* without a genomic sequence. RNA-seq gives more accurate gene expression data with relatively little technical variation compared to microarray and has been used for transcriptome profiling in various species with or without genome sequences, such as Zebrafish, *Chrysomya megacephala* and *Sedum alfredii* Hance [39–41].

In the present study, we characterized the global gene expression profiles of *S*. *baicalensis* across different organs (roots, stems, leaves and flowers) using Solexa/Illumina (San Diego, California, USA) high-throughput RNA sequencing technology. Candidate genes coding for enzymes involved in the biosynthesis of flavonoid were discovered. Furthermore, the differential expression models and simple sequence repeats (SSRs) associated with these genes were carefully analyzed. Our results proposed that RNA-seq could be an ideal method to obtain insights into the complex transcriptome of *S*. *baicalensis*, and these transcriptome datasets will provide a valuable resource for research on the regulation of flavonoid biosynthesis.

## Materials and Methods

### Plant Materials and RNA Preparation

Three-year-old S. *baicalensis* plants were grown in the experimental field of Beijing University of Chinese Medicine (Beijing, China) during the natural growing seasons. Leaves, stems, flowers and main roots were separately sampled at the bloom stage on July 8th, 2013 and frozen immediately in liquid nitrogen until RNA extraction. Total RNAs were isolated using TRIzol according to the manufacturer's protocols and then treated with RNase-Free DNase to remove residual genomic DNA contamination. The quality and quantity of RNA for RNA-seq sequencing libraries was assessed using the Agilent Technologies 2100 Bioanalyzer with an RNA integrity number (RIN) of more than 7.

### Library Construction and RNA-sequencing

The four libraries for transcriptome sequencing were prepared using Illumina’s kit following the manufacturer’s instructions and sequenced using the HiSeq2000 sequencing platform. In brief, all of the poly (A)-mRNA from the total RNA was purified and isolated using Magnetic Oligo(dT) beads. Then, the purified RNA was sheared to an average fragment size of 330 nt prior to cDNA synthesis. Subsequently, the short fragments were purified and ligated to sequencing adapters. Following agarose gel electrophoresis, suitable fragments were selected to be templates for PCR amplification, and the final PCR products were sequenced using Illumina HiSeq 2000 as 100-bp, paired-end reads.

### Assembly of Transcriptome

Each set of PE sequence reads was first processed with Trimmomatic[42] and FastQC (available from: http://www.bioinformatics.babraham.ac.uk/projects/fastqc/) to assess raw read qualities, remove low quality base pairs and sequence adapters. RNA-seq reads remaining after quality control were assembled using the Trinity (Release v2.0.6)[43], Velvet (version: 1.2.10)[44] and Oases (version: 0.2.08)[45] to obtain high-quality transcript sequences. We ran Trinity using the follow parameters:—min_contig_length 300,—KMER_SIZE 25,—min_kmer_cov 2,—group_pairs_distance 350,—path_reinforcement_distance 70,—min_glue 2 and other parameters set to default. Different k-mer lengths ranging from 31 to 95 were used for Velvet and Oases. Contigs obtained from Trinity, Velvet and Oases were merged together and then subjected to CD-HIT-EST[46] to remove redundancy and retain the longest possible contigs. Finally, the output sequences of CD-HIT-EST were clustered using TGICL[47] to produce longer and more complete consensus sequences with minimum and maximum overlap length of 40 and 90, respectively[48]. Such sequences were defined as unigenes in this study. To evaluate the accuracy of the assembled sequences (unigenes), SOAPaligner (Release 2.21, 02-14-2011) was employed to realign all the usable sequencing reads onto the unigenes and only positive unigenes were kept to downstream analyses.

### Functional Annotation

Functional annotation for assembled sequences was carried out through BLAST (ncbi-blast-2.2.27+) against a series of databases, including Swiss-Prot [49], InterPro [50], KEGG [51], NR [52], and NT [52] with a common significant threshold cut-off of E-value ≤1e-10^−6^. The functional categories of these assembled sequences were performed according to gene ontology (GO) annotations based on InterPro Go slims provided by Interproscan 5. The metabolic pathway analysis was accomplished with KEGG Automatic Annotation Server (KAAS) [51]. BBH (bi-directional best hit) method was used to search against the KEGG database and to obtain the KO (KEGG orthology) number and KEGG’s reference metabolic pathway for the assembled sequences. The SBH (the single-directional best hit) method also have been used as an alternative to avoid the loss of import genes including in the pathway of flavonoid biosynthesis.

### Identification of Simple Sequence Repeats (SSRs)

The source code for perl script as a stand-alone SSR identification tool was downloaded from http://www.gramene.org and modified to run in a batch mode. The motif-length for SSR analysis included dimers, trimers, tetramers, pentamers, hexamers, heptamers, octamers, nonamers and decamers. The minimum number of repeats for dimers and trimers was 9 and 6, respectively, and the minimum number of repeats for tetramers, pentamers, hexamers, heptamers, octamers, nonamers and decamers was 5.

### Gene Expression Analysis

The gene expression profiles were compared by mapping the RNA-seq reads with assembled sequences using Bowtie2 2.1.0 by the option “-N 0” to restrict 0 mismatches in seed alignment region [53]. The gene expression levels were measured and normalized as the reads per kilobase of transcripts model per million mapped reads (RPKM) using an EM algorithm (RSEM)[54]. Then, the differentially expressed genes were detected using a MA-plot-based method with random sampling model in the R statistical programming environment (edgeR)[55] that utilized a fold change of more than 4 and a *P* value of less than 0.001 as the threshold[56]. All differentially expressed genes were mapped to the GO database and compared to the whole transcriptome background for GO enrichment analysis. BiNGO [57] was used to calculate enrichment GO term by using custom annotation files from transcriptome based on the hypergeometric test (*P* <0.05)[58].

### Validation of Differentially Expressed Flavonoid Biosynthetic Genes (DEGs)

Six differentially expressed flavonoid biosynthetic genes were chosen and validated by real-time qPCR with gene-specific primers designed by Primer3 software (http://primer3.ut.ee/). A SuperScript III reverse transcription kit (Invitrogen, USA) was used to convert mRNAs into cDNAs, and real-time quantification was performed using the Chromo4 Real-time PCR Instrument (MJ, USA) and SYBR Green method (Invitrogen, S-7567). The PCR was conducted in a 20 μl volume containing 4 μl diluted cDNA, 250 nM forward primer, 250 nM reverse primer, and 1×SYBR Premix Ex Taq II (TaKaRa) using the following conditions: 95°C for 3 min, 40 cycles of 95°C for 15 sec, 59°C for 15 sec and 72°C for 15 sec. The *GAPDH* gene was used as an internal standard for data normalization. Ct values were determined based on three technical replicates of each sample and were transformed into relative quantification data using the 2^-ΔΔCt^ relative quantitative method [59]. All data were showed as the mean ± SD after normalization.

## Results

### Sequencing and *de novo* Assembly

**Fig 1:**
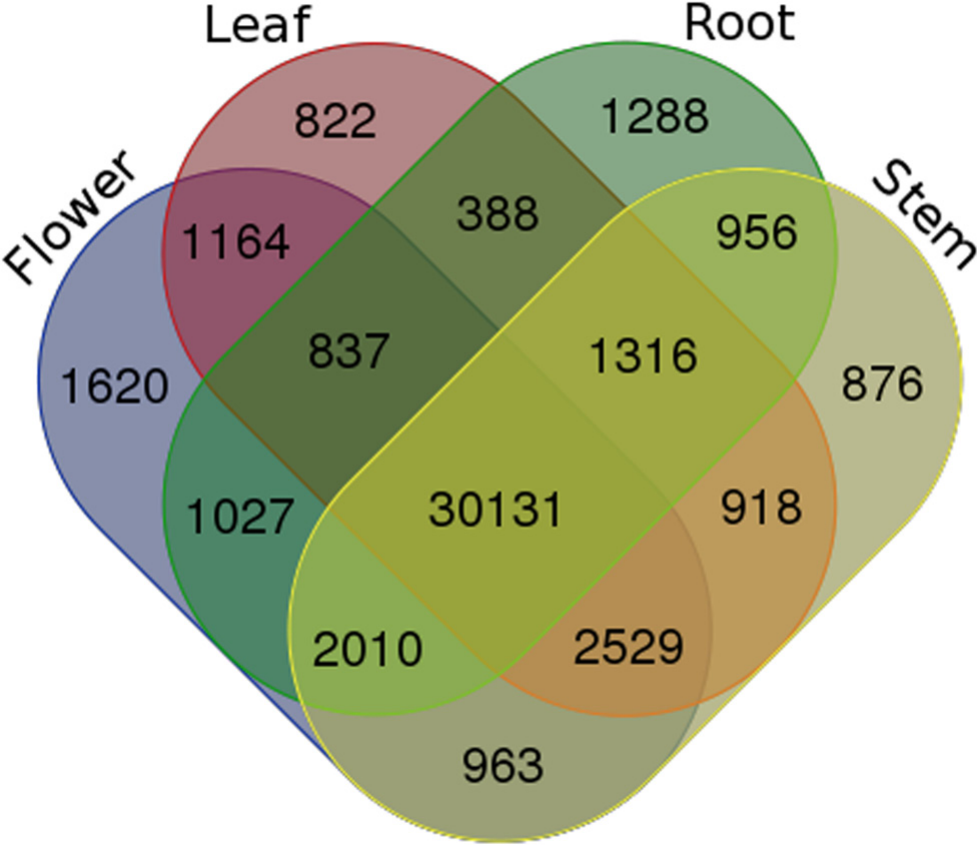
Venn diagram of unigenes in the roots, stems, leaves and flowers of *S*. *baicalensis*.

To characterize the transcriptome of *S*. *baicalensis* and generate expression profiles, RNAs from roots, stems, leaves and flowers were extracted and processed for high-throughput Illumina/Solexa paired-end sequencing. The Illumina/Solexa transcriptome sequencing was conducted and produced approximately 91 million paired-end reads of 100-nt in length (Table 1). Total numbers of paired-end reads from the sequencing libraries of roots, stems, leaves and flowers were 22,817,910, 22,441,855, 22,525,547 and 23,695,448, respectively. Trinity, Velvet and Oases assembly software programs were used for *de novo* assembly and yielded 49,507 unigenes. There were 37,953, 39,699, 38,105, and 40,281, unigenes in roots, stems, leaves and flowers respectively. The four organs shared 30,131unigenes, likely including housekeeping genes which might play non-redundant roles in *S*. *baicalensis*. The number of unigenes only can be found in each organ was 1,288 for the roots, 876 for the stems, 822 for the leaves, 1,620 for the flowers (Fig 1). Approximately 23,813 (48.1%) of the total unigenes had lengths of more than 1 kb and 33,151 (71.6%) unigenes with reads per kilobase of transcripts model per million mapped reads (RPKM) of more than 3 in at least one of the organs. RNA-seq data has been deposited in the SRA database under accession number SRR1605127.

**Table 1:** Overview of Illumina sequencing and transcript assembling obtained from four RNA-seq libraries.

Sample	Roots	Stems	Leaves	Flowers	Total
Reads (paired-end)	22 817 910	22 441 855	22 525 547	23 695 448	91 480 760
Average Length	100 (base)	100 (base)	100 (base)	100(base)	100 (base)
Total No. of bases	4 563 582 000	4 488 371 000	4 505 109 400	4 739 089 600	18 296 152 000
Mapped reads^a^	40 385 914	38 670 200	35 825 402	42 358 298	157 239 814
Percentage^b^	88.5%	86.2%	79.5%	89.4%	85.9%
RPKM>3^c^	21 699	21 642	18 970	20 929	30 545

^a^ reads that map to assembled unigene sequences

^b^ percentage of reads that map to assembled unigene sequences

^c^RPKM: reads per kilobase of unigene model per million mapped reads

### Functional Annotation

In total, more than 70% of transcripts were annotated in at least one of the public databases (Swiss-Prot, InterPro, KEGG, NR and NT). The rest not annotated appeared to be either *S*. *baicalensis*-specific genes or homologous genes with unknown functions in other species. Gene Ontology (GO) term analysis was subsequently used to classify the genes into three GO categories (biological process, cellular component and molecular function). In total, 21,172 unigens have been mapped to 1,940 GO terms (biological process, 757; cellular component, 243; molecular function, 940, S1 Table). The largest cellular component for *S*. *baicalensis* represented those integral membranes, i.e., nucleus and plasma membrane. The majority of biological processes were involved in transcription, regulation of transcription and proteolysis, and most of the molecular functions were associated with ATP, metal-ion and DNA binding. One candidate baicalinase gene, three candidate baicalein 7-O-glucuronosyltransferases genes, four candidate 6-hydroxylase genes, and one candidate 8-O-methyltransferase gene for the biosynthesis of wogonin, wogonoside, baicalin, and baicalein were recognized based on the annotation results.

KEGG pathway database is a database that collected manually drawn pathway maps on molecular interaction and reaction networks, such as biosynthesis of secondary metabolites [51]. To identify the biosynthesis pathways of active compounds in *S*. *baicalensis*, we mapped all of the assembled sequences to the KEGG pathway database. The KO (reference pathway) number of the transcriptome was determined according to KEGG annotation. A total of 13,627 unigenes were assigned to 3,810 KEGG genes and found to be involved in 579 different pathways (S2 Table). “Ribosome” was the dominant type, followed by “Biosynthesis of amino acids”, “Spliceosome”, “Oxidative phosphorylation”, “Purine metabolism”, “Carbon metabolism”, “RNA transport”, “Protein processing in endoplasmic reticulum”, “Huntington's disease”, “Pyrimidine metabolism”.

### Gene Expression Analysis and Identification of DEGs in Different Organs

To reveal the expression profiling, we sequenced and calculated the global gene expression of four different organs including roots, stems, leaves, and flowers. The results revealed that 88.5%, 86.2%, 79.5%, and 89.4% of the sequencing reads from roots, stems, leaves and flowers library could be mapped back to the assembled sequences, respectively. Subsequently, raw counts for each assembled sequence were calculated based on the alignment files, and the gene expression levels were measured and normalized as RPKM. The average RPKMs in roots, stems, leaves and flowers were 18.4, 17.1, 17.5, and 16.7, respectively, and in total, 21,699, 21,642, 18,970 and 20,929 unigenes were detected with RPKM of more than 3 in the libraries of roots, stems, leaves and flowers, respectively. These results demonstrated that RNA-seq provided high resolution of gene expression.

**Fig 2:**
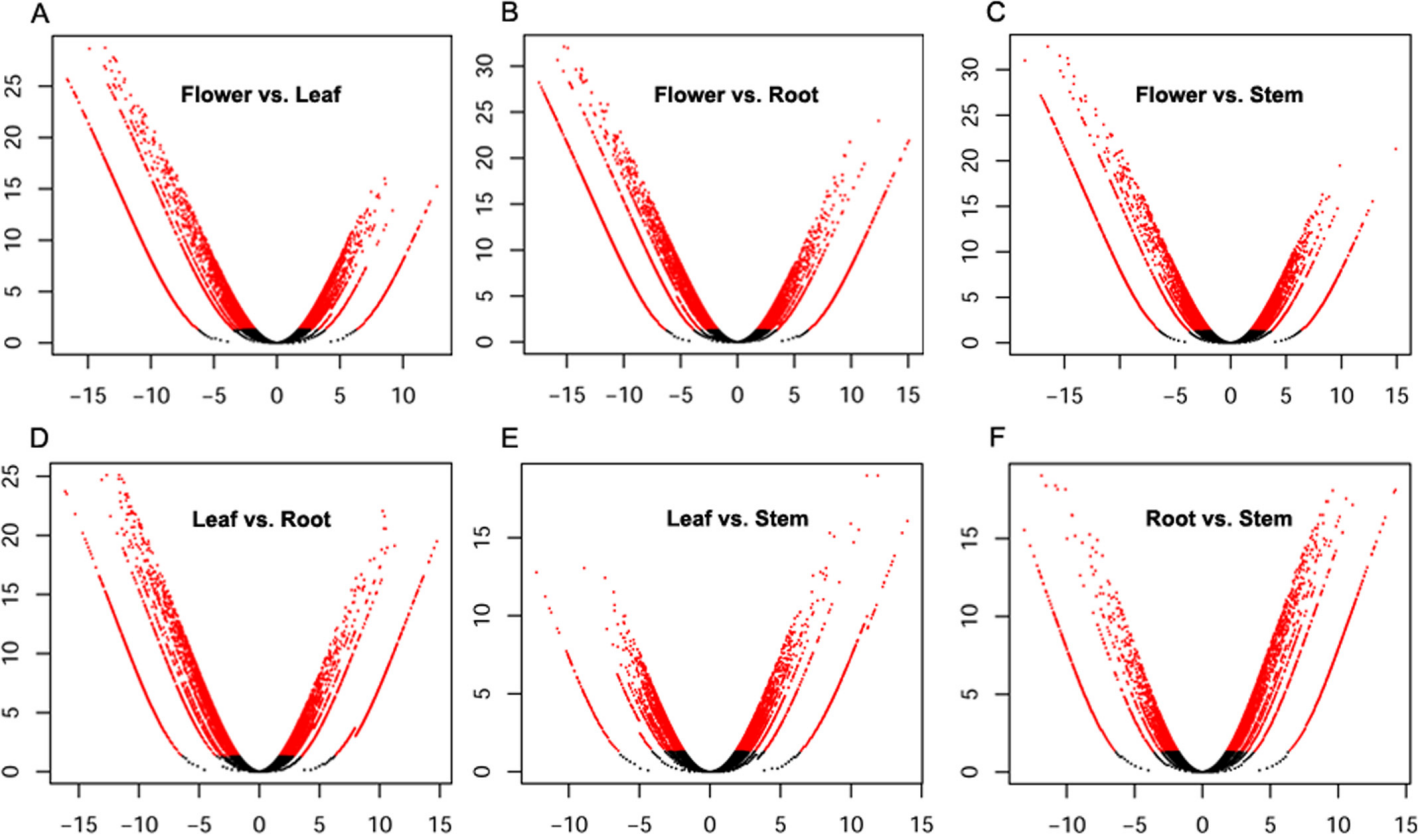
Volcano plots of differentially expressed genes based on pair-wise comparison analyzed by RNA-sequencing. The x-axis and y-axis show the fold changes on a log2 scale and the *P* on a –log10 scale, respectively. Genes differentially expressed with >4-fold and P<0.001 are presented in red.

**Fig 3:**
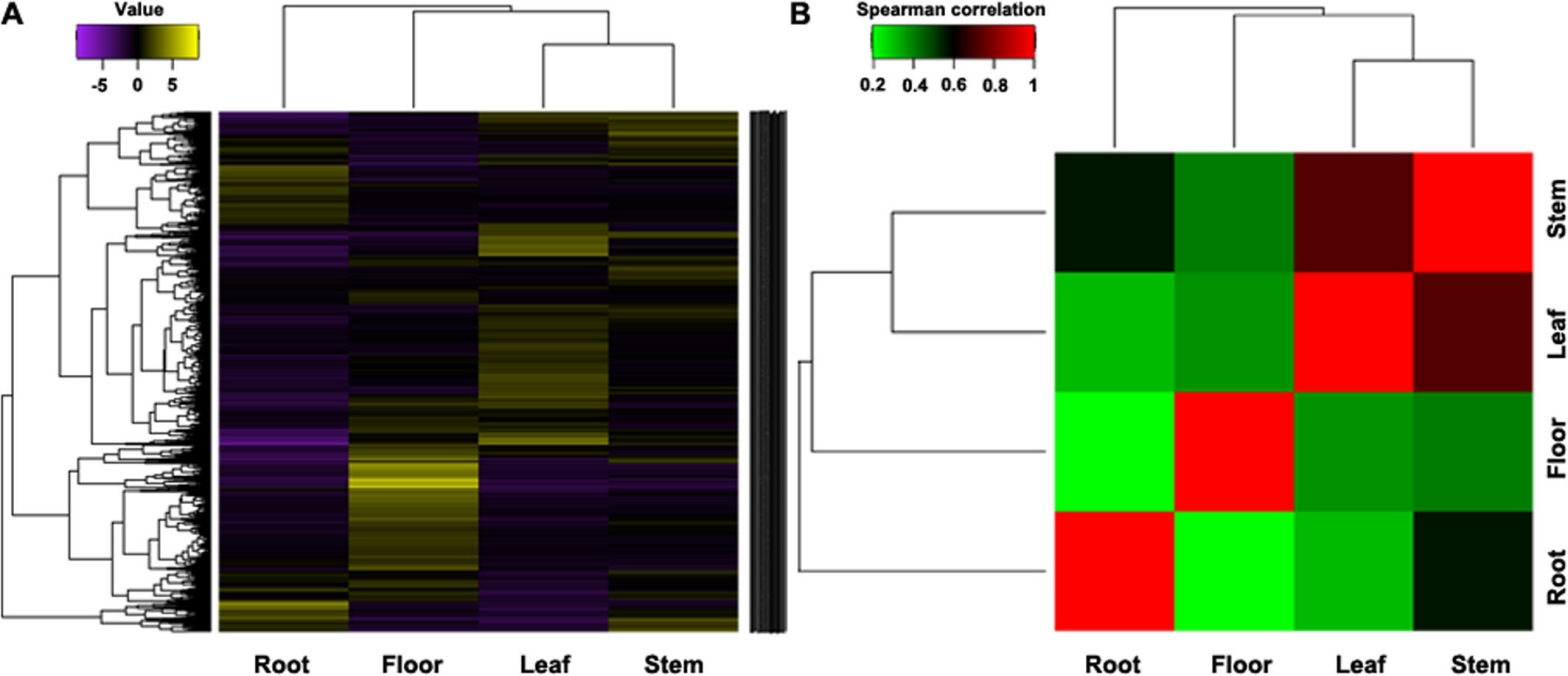
Clustering of differentially expressed genes from four different organs of *S*. *baicalensis* generated by hierarchical clustering.

To obtain the global expression changes in different organs, we applied the MA-plot-based method with random sampling model in the R statistical programming environment to identify the differentially expressed genes by pair-wise comparisons across different organs[55]. To identify differentially expressed genes, we used a rather strict threshold of fold change of >4 and a *P*<0.001 to guarantee that only strong alterations in the gene expression levels were reported based on the pair-wise comparisons. Ultimately, thousands of genes showed significant differential expression between various organs (Fig 2). In total, 7,647 up-regulated genes and 5,891 down-regulated genes between at least two organs were found (S3 Table). The differential expression patterns of genes across four organs were clustered into six different clusters. The gene expression in leaves and stems were clustered together and separated from roots and flowers (Fig 3). We focused on the up-regulated genes in roots which could produce bioactive constituents. In total, there were 469 genes up-regulated in roots comparing with stems, leaves and flowers. The GO terms for these genes belonged to molecular function were enrichment for flavonol 3-O-glucosyltransferase activity and quercetin 4'-O-glucosyltransferase activity, respectively. Further study of the candidate genes will provide a better understanding of genes that were overrepresented in different organs of *S*. *baicalensis*.

### Candidate Genes Coding for Enzymes Involved in the Biosynthesis of Flavonoid

**Fig 4:**
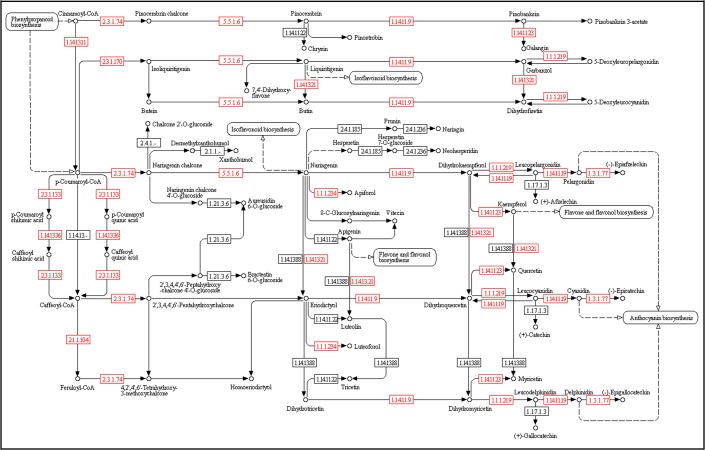
Metabolic pathway for the biosynthesis of flavonoids in *S*. *baicalensis* according to KEGG annotation. 12 enzymes from the transcriptome sequences dataset are marked in red boxes.

Flavonoids, which are synthesized from phenylpropanoid derivatives by condensation with malonyl-CoA [60], are a major class of plant secondary metabolites with various biological activities including pigments and antioxidant activity. Presently, 15 homologous enzymes encoding genes have been found to be implicated in flavonoid biosynthesis pathway in plants. And 12 of them have been discovered in this study: chalcone synthase (K00660, CHS); chalcone isomerase (K01859, E5.5.1.6); naringenin 3-dioxygenase (K00475, E1.14.11.9); flavonol synthase (K05278, FLS); trans-cinnamate 4-monooxygenase (K00487, CYP73A); flavonoid 3'-monooxygenase (K05280, E1.14.13.21); leucoanthocyanidin dioxygenase (K05277, E1.14.11.19); shikimate O-hydroxycinnamoyltransferase (K13065, E2.3.1.133), coumaroylquinate (coumaroylshikimate) 3'-monooxygenase (K09754, CYP98A3), bifunctional dihydroflavonol 4-reductase/flavanone 4-reductase (K13082, DFR), caffeoyl-CoA O-methyltransferase (K00588, E2.1.1.104), and anthocyanidin reductase (K08695, ANR) (Fig 4). Except chalcone isomerase and coumaroylquinate (coumaroylshikimate) 3'-monooxygenase, the other nine enzymes encoding genes have been found to be up-regulated or down-regulated in at least one organ. Five genes encoding naringenin 3-dioxygenase, leucoanthocyanidin dioxygenase, flavonol synthase, flavonoid 3'-monooxygenase and bifunctional dihydroflavonol 4-reductase/flavanone 4-reductase were up-regulated in leaves and down-regulated in roots, flowers, and stems. The gene coding caffeoyl-CoA O-methyltransferase was up-regulated in roots and stems but down-regulated in leaves and stems.

Four major flavonoids, i.e., wogonin, wogonoside, baicalin, and baicalein, make up approximately 1.3%, 3.55%, 5.41% and 10.11% of the dry material of *S*. *baicalensis*, respectively [61]. We screened the transcriptome of *S*. *baicalensis* and identified one baicalin-beta-D-glucuronidase (EC Number: 3.2.1.167; baicalinase), which can directly hydrolyze baicalin/wogonoside into baicalein/wogonin, respectively. We also found three baicalein 7-O-glucuronosyltransferases (EC Number: 2.4.1.253) specific for UDP-D-glucuronate as a sugar donor and flavones with an ortho- substitution at the 7-OH group of molecules such as baicalein, scutellarein and wogonin. The transcriptional level of baicalinase was abundant in leaves and flowers, whereas the transcriptional level of baicalein 7-O-glucuronosyltransferases were abundant in roots, suggesting that the content of baicalin/wogonin and baicalein/wogonoside could be differentially regulated in different organs. The above-mentioned, differentially expressed genes in roots, stems, leaves and flowers according to the Solexa/Illumina sequencing results were validated by qRT-PCR. The forward and reverse primers used for qRT-PCR validation are listed in S4 Table. The expression profiles from the qRT-PCR results (S1 Fig) were nearly in complete agreement with those obtained by the RNA-seq data. This high correlation supported the reliability of the RNA-seq results.

### Detection of Simple Sequence Repeats (SSRs)

Microsatellites, also known as SSRs, are nucleotide sequences that are repeated in tandem and can provide an efficient tool to link phenotypic and genotypic variation [62, 63]. This study provided us with high-throughput identification and screening of SSR candidate markers from large amounts of assembled sequences. In total, 6,901 SSR markers were identified in the *S*. *baicalensis* transcriptome (S5 Table). The number of repeat times for a simple sequence ranged from 5 to 35, with 9 being the most frequent followed by 6 and 10 repeats. SSR types were categorized by repeat type: dimer motifs were the most abundant and accounted for 68.5% of all characterized SSRs, followed by trimers (27.0%), and tetramers (1.9%). The predominant dimer repeat motifs were GA, CT, AG, and TC. The predominant trimer repeat motifs were TTC and TCT. There were seven unigenes in the flavonoid biosynthesis pathway that had candidate SSRs. The first large-scale survey of microsatellites derived from *S*. *baicalensis* provided abundant molecular-assisted selection markers for further investigation.

## Discussion

*Scutellaria baicalensis* Georgi (Huang-qin) is a perennial herb of the Lamiaceae family. The root of this herb (Radix Scutellariae) is extensively employed in traditional Chinese medicine and modern herbal prescriptions. The *S*. *baicalensis* natural flavonoids, especially their glycosides have diverse biological activities and *S*. *baicalensis* has been proposed as an excellent model system for continued research of useful medicinal compounds[64]. In this study, high-throughput RNA-Seq technique was used to characterize the transcriptome of *S*. *baicalensis* from four different organ samples (roots, stems, leaves, and flowers). Altogether, a transcriptome-wide analysis of the high-throughput RNA-Seq results revealed the presence of 49,507 unigenes. The analysis of the *S*. *baicalensis* transcriptome based on deep transcriptome sequencing provided first remarkable insights into the transcriptional profiling of the important medical plant, and it also offers new knowledge for understanding the biosynthesis of flavonoid in *S*. *baicalensis*. More importantly, candidate key enzymes (baicalinase, 7-O-glucuronosyltransferases, 6-hydroxylase, 8-O-methyltransferase) for the formation of wogonin, wogonoside, baicalin, and baicalein were recognized preliminarily. A total of 6,901 SSR markers in the *S*. *baicalensis* transcriptome were first identified in our study. The identification of SSRs markers linked to genes involved in the pathway of flavonoid biosynthesis constitute a valuable resource of ideal markers for the molecular breeding of this important traditional herbal medicine.

Flavonoids isolated from the roots of *S*. *baicalensis* are the major components used for the treatment of disease and were found to compose most of the dry material of *S*. *baicalensis* [61, 65]. It was confirmed that the active components were four major flavonoids: baicalin and its aglycone baicalein, wogonoside and its aglycone wogonin [66, 67]. Baicalein, wogonin, and baicalin, with anti-cancer, anti-oxidative, anti-inflammatory, anti-bacterial and anti-viral properties [68], had shown effects on human prostate cancer cell growth and survival [65, 69]. Wogonoside inhibits LPS-induced angiogenesis both *in vitro* and *in vivo*, and might have a therapeutic potential for the diseases associated with the development of both inflammation and angiogenesis progress [70]. Our transcriptome revealed 54 unigenes that encode 12 key enzymes involved in the flavonoid biosynthesis pathway. Here, we focused on the key enzymes directly linked on the biosynthesis of the four mentioned active components. Three unigenes coding flavonol synthase have been recognized in our study, whereas no flavone synthase gene has been discovered. This implied that, in *S*. *baicalensis*, 3,5,7-Trihydroxyflavone was the precursor of baicalein, wogonin, baicalin, and wogonoside. Previous research showed that baicalein (5,6,7-trihydroxyflavone)/wogonin (5,7-dihydroxy-8-methoxyflavone) can be hydrolyzed from baicalin (baicalein 7-O-glucuronide)/wogonoside (wogonin 7-glucuronide) *in vivo* by cleavage of the glycoside moiety with beta-D-Glucuronidase (baicalinase, GUS [EC 3.2.1.31]) [71, 72]. Similarly, baicalein/wogonin can be converted into baicalin/wogonoside using UDP-glucuronate acid as a sugar donor by catalyzing with baicalein 7-O-glucuronosyltransferase (UBGAT, EC 2.4.1.253) [73]. In this study, we identified one baicalinase and three baicalein 7-O-glucuronosyltransferases that were involved in the transformation between baicalin/wogonoside and baicalein/wogonin according to homology analysis. Unlike methyltransferase, the flavonoid glycosyltransferase doesn’t have position and substrate specificities[74]. Chiou demonstrated that one type of flavonoid glucosyltransferase can make various flavonoids as substrates. Moreover, this glucosyltransferase can glycosylate the 7-OH group substrate when the 3-OH group was not available[34]. From all of the differentially expressed genes related to flavonoid biosynthesis and the formation of baicalein, wogonin, baicalin, and wogonoside, we found several enzymes showed organ-specific expression, suggesting that the content of baicalein and baicalin could be regulated by these enzymes in different manners. We will pay attention to these flavonoid biogenesis genes in future analyses of bioactive compounds. The transcriptome information for this biosynthesis provides a solid foundation for further characterization of the regulation of the biosynthesis of baicalein and baicalin, and biochemical and physiological studies on the candidate biosynthesis genes of baicalein and baicalin will be implemented in the future.

## Supporting Information

S1 Fig Real-time PCR validation of differentially expressed genes that involved in flavonoid biogenesis genes. F, L, R, S referred to flower, leaf, root, stem respectively.
(TIF)

S1 Table The annotation results of gene ontology (GO) term analysis using InterProScan 5. (XLSX)

S2 Table KEGG pathways identified by KEGG automatic annotation server (KAAS). (XLSX)

S3 Table The number of up- and down-regulated DEGs based on pair-wise comparison. (DOC)

S4 Table Primers of six selected genes related to the pathways of flavonoid biosynthesis for RT-PCR. (DOC)

S5 Table The summary of occurrence of simple sequence repeats (SSRs) in *S*. *baicalensis*. (XLSX)
