# Supplementary figures and images for: Deep Sequencing of the Scutellaria baicalensis Georgi Transcriptome Reveals Flavonoid Biosynthetic Profiling and Organ-Specific Gene Expression
Source: PLoS One. 2015 Aug 28;10(8):e0136397. doi: 10.1371/journal.pone.0136397 (PMC4552754; doi:10.1371/journal.pone.0136397)

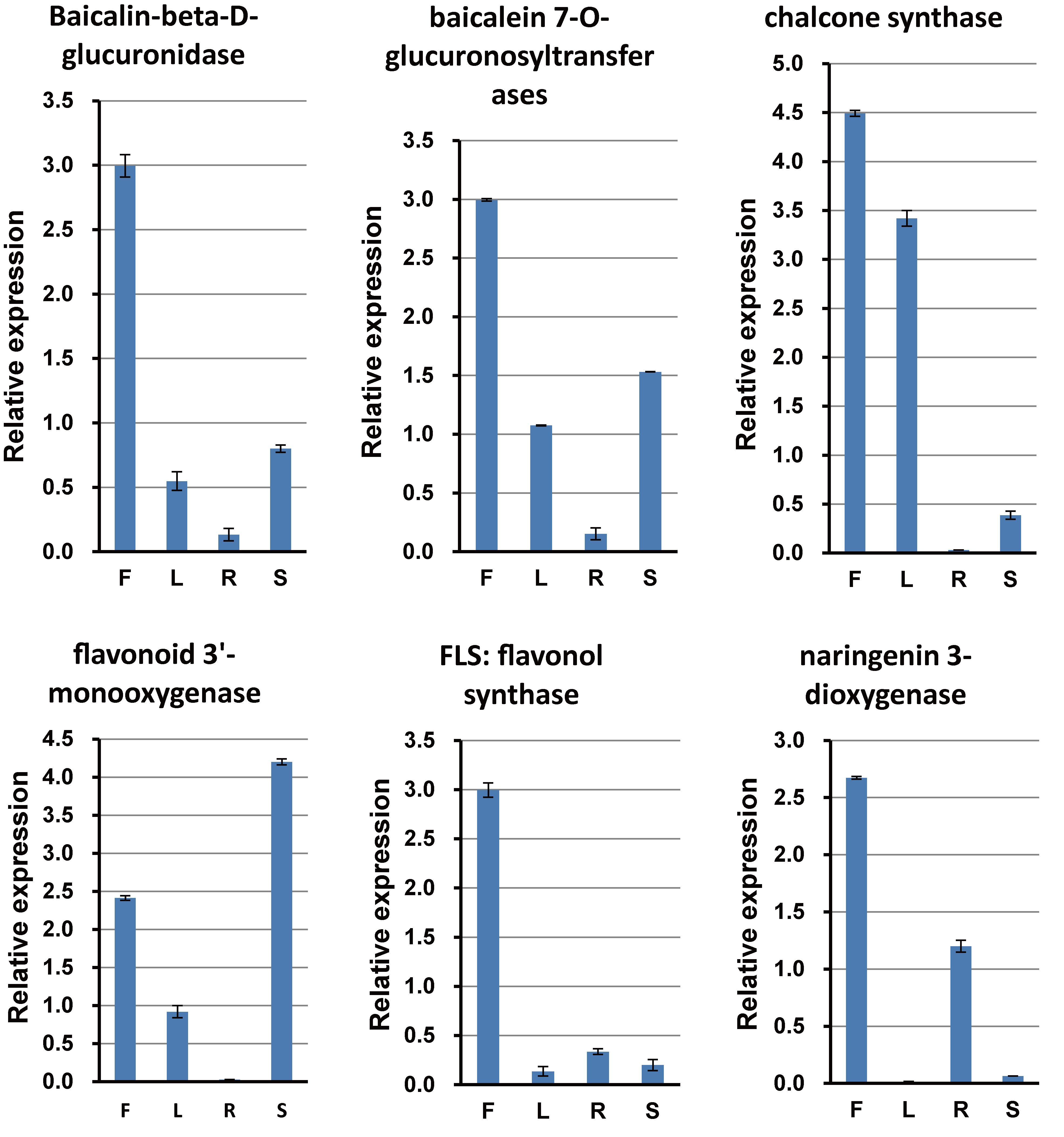

Supplement: S1 Fig — F, L, R, S referred to flower, leaf, root, stem respectively. (TIF) [file pone.0136397.s001.tif]
