# Supplementary material for: Deep Sequencing of the Scutellaria baicalensis Georgi Transcriptome Reveals Flavonoid Biosynthetic Profiling and Organ-Specific Gene Expression
Source: PLoS One. 2015 Aug 28;10(8):e0136397. doi: 10.1371/journal.pone.0136397 (PMC4552754; doi:10.1371/journal.pone.0136397)
Supplement: S3 Table — (DOC) [file pone.0136397.s004.doc]

**Table S3. The number of up- and down-regulated DEGs based on pair-wise comparison**

| Pair-wise comparisons | Up-regulated | Down-regulated |
| --- | --- | --- |
| flowers VS leaves | 2,569 | 1,677 |
| flowers VS roots | 3,724 | 1,510 |
| flowers VS stem | 2,117 | 1,458 |
| leaves VS roots | 3,662 | 1,746 |
| leaves VS stem | 941 | 1,086 |
| roots VS stem | 854 | 2,185 |
