# Supplementary material for: Deep Sequencing of the Scutellaria baicalensis Georgi Transcriptome Reveals Flavonoid Biosynthetic Profiling and Organ-Specific Gene Expression
Source: PLoS One. 2015 Aug 28;10(8):e0136397. doi: 10.1371/journal.pone.0136397 (PMC4552754; doi:10.1371/journal.pone.0136397)
Supplement: S4 Table — (DOC) [file pone.0136397.s005.doc]

**Table S4. Primers of six selected genes related to the pathways of flavonoid biosynthesis for RT-PCR**

| Genes | primers | Sequences |
| --- | --- | --- |
| Baicalin-beta-D-glucuronidase | Forward | GTGAGAGAGGACACGCAGAT |
|  | Reverse | ATGGTTGGGCACTTGGAAAC |
| baicalein 7-O-glucuronosyltransferases | Forward | CGGAAATGCATACCCTTGGG |
|  | Reverse | ACGGAAAGCGACGATCAAAG |
| chalcone synthase | Forward | GAAGAACAGAGCACGCTAGC |
|  | Reverse | GCATTTGGACCAGGGATCAC |
| flavonoid 3'-monooxygenase | Forward | CTCTTCCTGTCTGACGTGGT |
|  | Reverse | TGAAGATGGGGTTGGCTCAT |
| flavonol synthase | Forward | ACCATCCTCGTTCCCAATCA |
|  | Reverse | CCATGAACACAGGCCAAGAC |
| naringenin 3-dioxygenase | Forward | ACAACAAGTTCAGCGACGAC |
|  | Reverse | GAGGAGGCAATGCGAAGAAG |
| GAPDH | Forward | AGGCCCTCGACAATACCAAA |
|  | Reverse | CCCATCTTAAGGGTGGAGCA |
